# Predictive Value of Optical Coherence Tomography Biomarkers in Patients with Persistent Diabetic Macular Edema Undergoing Cataract Surgery Combined with a Dexamethasone Intravitreal Implant

**DOI:** 10.3390/bioengineering12050556

**Published:** 2025-05-21

**Authors:** Giuseppe Fasolino, Maryam Lazaar, Domenico Giovanni Della Rocca, Silke Oellerich, Sorcha Ní Dhubhghaill

**Affiliations:** 1 Department of Ophthalmology, University Hospital Brussels, Laarbeeklaan 101, 1090 Jette, Belgium; 2 Department of Medicine and Pharmacy, Vrije Universiteit Brussel (VUB), 1090 Brussels, Belgium

**Keywords:** cataract, dexamethasone, diabetic retinopathy, diabetic macular edema, optical coherence tomography, intravitreal corticosteroids

## Abstract

**Background:** Diabetic macular edema (DME) is the most common cause of vision loss among diabetic patients. The first-line treatments for DME are anti-vascular endothelial growth factor (VEGF)-drugs, while intravitreal steroids are generally reserved for second-line treatment. Limited data exist on the role of optical coherence tomography (OCT) biomarkers as predictors of success in non-responders to anti-VEGF treatment undergoing simultaneous cataract surgery and dexamethasone intravitreal implant (DEX-I). **Methods:** This study was designed as a retrospective analysis of patients with DME who were refractory to anti-VEGF treatment but underwent cataract surgery and received a DEX-I at the time of surgery. All procedures were performed between May 2021 and February 2024. The best-corrected visual acuity (BCVA) and central subfoveal thickness (CST) were recorded at baseline and at 1 week, 1 month, and 3 months. The following OCT-based biomarkers were also collected: ellipsoid zone (EZ) integrity, disorganization of the retinal inner layers (DRIL), CST, and hyperreflective foci (HRF). Correlations between the baseline biomarkers and the anatomical outcome were analyzed using linear mixed models (LMMs). **Results:** Eleven patients (eighteen eyes) met the inclusion criteria. The mean CST decreased significantly from 469.4 ± 53.8 µm at baseline, to 373.1 ± 34.7 µm at 1 week (*p* = 0.002) and 354.4 ± 24.1 µm at 1 month (*p* = 0.011). The mean BCVA improved significantly from 0.47 LogMAR to 0.33 LogMAR at 1 week (*p* = 0.001), 0.23 LogMAR at 1 month (*p* < 0.001), and 0.25 LogMAR at 3 months (*p* < 0.001). Baseline predictors significantly influencing CST included the presence of DRIL, a disrupted/absent EZ, and a higher CST. **Conclusions:** The administration of DEX-I for DME refractory to anti-VEGF treatment in patients undergoing cataract surgery promoted functional improvements persisting longer than the anatomical ones. Patients presenting with DRIL, disrupted EZ, and higher CST at baseline may be better candidates for the combination of DEX-I and cataract surgery.

## 1. Introduction

Diabetes mellitus (DM) is a systemic disease which significantly contributes to the development of several comorbidities (e.g., chronic kidney disease, cardiovascular disease, stroke) [1]. Given the nature of the delicate retinal vasculature, DM is also detrimental to the eye and has been linked to several conditions, including retinal damage and cataract formation [2]. Diabetic macular edema (DME) is the most common cause of vision loss among DM patients [3].

The prevalence of DME in the diabetic population varies between 1.4% and 12.8%; although its exact pathogenesis is not entirely clear, it is believed to be multifactorial and is influenced by several factors (e.g., diabetes type, ethnicity and duration) [4]. From a pathophysiological standpoint, persistent high blood glucose levels can trigger a series of biochemical responses, including increased oxidative stress, inflammation and blood-retinal barrier disruption [3]. The induced inflammation leads to a breakdown of the vascular barrier. This process is associated with increased levels of vascular endothelial growth factor (VEGF) as well as other interleukins (e.g., IL-1β, MCP-1, IP-10, IL-6, IL-8), as has been shown in the aqueous humor samples [5]. Given these pathological mechanisms, the adoption of anti-VEGF drugs to counter the increased vascular permeability has shown encouraging outcomes, which justifies the use of these medications as the first-line treatments for DME.

Intravitreal steroids are another therapeutic alternative but are generally considered as a second-line strategy due to their relatively higher risk of increased intraocular pressure (IOP) and cataract [6]. Dexamethasone intravitreal implant (DEX-I) is a corticosteroid treatment indicated for adults with vision disorders due to DME, especially when they are refractory to non-steroidal treatments, have contraindications for anti-VEGF treatment or are pseudophakic. Patients who have persistent retinal fluid despite receiving consistently intravitreal injections are referred to as persistent diabetic macular edema (pDME). However, there is no clear consensus on the definition of pDME, leading to variability in treatment duration, injection frequency, and response assessment. Sorour et al. suggest a duration of 6 months as the most reasonable criterion, as the number of anti-VEGF injections depends on the drug used [7]. In this study, we define pDME as a refractory increase in DME despite at least 6 months of anti-VEGF treatment.

The effectiveness of DME treatments can vary widely among patients; therefore, the identification of predictive factors would be of immense clinical value in identifying the DM subpopulation that benefits the most from this strategy. Previous research has explored predictive optical coherence tomography (OCT) biomarkers in DME patients treated with intravitreal corticosteroids [8]. These biomarkers are not only useful for diagnosing and monitoring DME progression but also to provide prognostic information regarding the disease progression and response to treatment [6]. The aim of this study was to describe the changes in OCT biomarkers in patients with pDME unresponsive to anti-VEGF injections who underwent simultaneous cataract surgery and DEX-I.

## 2. Materials and Methods

### 2.1. Study Design

This study was designed as a retrospective analysis of patients with DME who were refractory to anti-VEGF treatment but underwent cataract surgery and received a DEX-I at the time of surgery. All procedures were performed between May 2021 and February 2024. Patients were included in the study if they fulfilled the following criteria: (I) age ≥18 years; (II) history of type 1 or 2 DM; (III) controlled blood pressure (≤130/80 mmHg); (IV) visually significant cataract (diagnosed by slit lamp biomicroscopy); (V) non-proliferative diabetic retinopathy or proliferative diabetic retinopathy previously treated with laser photocoagulation; (VI) clinically significant macular edema (CST ≥ 300 μm). The exclusion criteria were as follows: (I) treatment of DME with intravitreal anti-VEGF within 3 months before surgery; (II) treatment with intravitreal corticosteroids before inclusion in the study; (III) presence of uveitis, neovascular glaucoma, uncontrolled glaucoma, retinal vein occlusion; (IV) intraoperative complications.

Data were collected at baseline, 1 week, 1 month, and 3 months after surgery. This follow-up schedule was chosen to assess the effect of a single dose DEX implant given during cataract surgery. The 3-month time point was selected because it matches the period when the implant has its strongest effect, usually between 60 and 90 days [9]. Although the DEX implant can release corticosteroids for up to six months, a second treatment is often needed earlier in clinical practice, typically around five months after the first injection [10].

The study complied with the Declaration of Helsinki and was approved by the Ethical Committee of the University Hospital of Brussels (protocol number: EC-2024-134).

### 2.2. Surgical Procedure

All patients underwent phacoemulsification under topical anesthesia, through a 2.4 mm incision on the axis of the corneal incision. Briefly, a dispersive ophthalmic viscosurgical device was injected in the anterior chamber, a 5.5 mm capsulorhexis was performed followed by dismantling of the nucleus using the “divide and conquer” technique. Then, a hydrophilic acrylic intraocular lens was implanted into the capsular bag followed by removal of the OVD closure of the wounds and intracameral injection of cefuroxime. At the end of cataract surgery, DEX-I was administered intravitreally via pars plana, directly into the inferotemporal quadrant [11].

### 2.3. Data Collection

Clinical data on age, gender, type and duration of diabetes, as well as preoperative hemoglobin A1c (HbA1c) values were extracted from electronic medical records. In addition, we collected and analyzed data from the ophthalmological examination, which included a microscopic examination of the anterior eye segment, intraocular pressure (IOP), best corrected visual acuity (BCVA) and an OCT. Snellen fractions were converted to logMAR for statistical analysis. The differences in the number of letters per line were not taken into account.

OCT scans taken during standard follow-up appointments were used. Spectral domain OCT imaging was performed using the Spectralis system (Heidelberg Engineering, software version 1.10.0.0). Assessment of the OCT-biomarkers was based on the scan showing the most pronounced morphological abnormalities. The raster scan comprised 19 consecutive B-scans and a macular map centered on the fovea. The Spectralis software (version 1.10.0.0), integrated within the system, automatically generated measurements for the CST across the nine subfields from the Early Treatment Diabetic Retinopathy Study (ETDRS) grid.

The presence of several morphologic features was assessed, including hyperreflective foci (HRF), disorganization of the retinal inner layers (DRIL), central subfoveal thickness (CST), and the integrity of the ellipsoid zone/external limiting membrane (EZ/ELM). HRF were identified as circular, hyperreflective dots within the retina and a manual count was performed to determine their number. To exclude hard exudates and microaneurysms, only foci meeting specific morphological criteria were included in the analysis. These criteria included the following: (I) reflectivity resembling that of the nerve fiber layer; (II) absence of back-shadowing; and (III) diameter less than 30 μm. Scans were then categorized into two groups (high HRF/low HRF) based on the average number of HRFs, using the number of 30 as a cut-off value as in a previous study [12].

DRIL was characterized by the absence of clear boundaries between the ganglion cell layer-inner plexiform layer complex, the inner nuclear layer, and the outer plexiform layer within the central fovea. It was categorized as absent or present [12]. The assessment of EZ and ELM integrity relied on the visibility and continuity of the first and second hyperreflective bands among the four outermost retinal layers in OCT, respectively. Integrity was classified as ‘intact’ when these bands were clearly visible and continuous, ‘disrupted/absent’ when they were partially visible or when they were completely lost [12].

### 2.4. Statistical Analysis

Linear Mixed Models (LMMs) were selected because they can manage missing data points and handle repeated measures. The dataset included 18 eyes of 11 patients, so for 7 patients, both eyes were included. The models included fixed effects for time, the biomarker, and their interaction. The subjects were included as random effects to account for repeated measures. The small sample size limited interaction analyses between biomarkers. *p*-values < 0.05 were considered statistically significant. SPSS (version 29.0.2.0) for macOS was used for the analyses.

## 3. Results

Eighteen eyes of eleven patients with pDME who underwent both cataract surgery and intravitreal DEX-I met the criteria for inclusion. Baseline demographic characteristics are reported in Table 1. The mean age of the patients was 62.7 years (±9.7 years) with a mean history of diabetes of 19.4 years (±9.1 years). All patients that met inclusion were male and all patients underwent follow-up examination at 1 week, 1 month, and 3 months.

**Figure 1:**
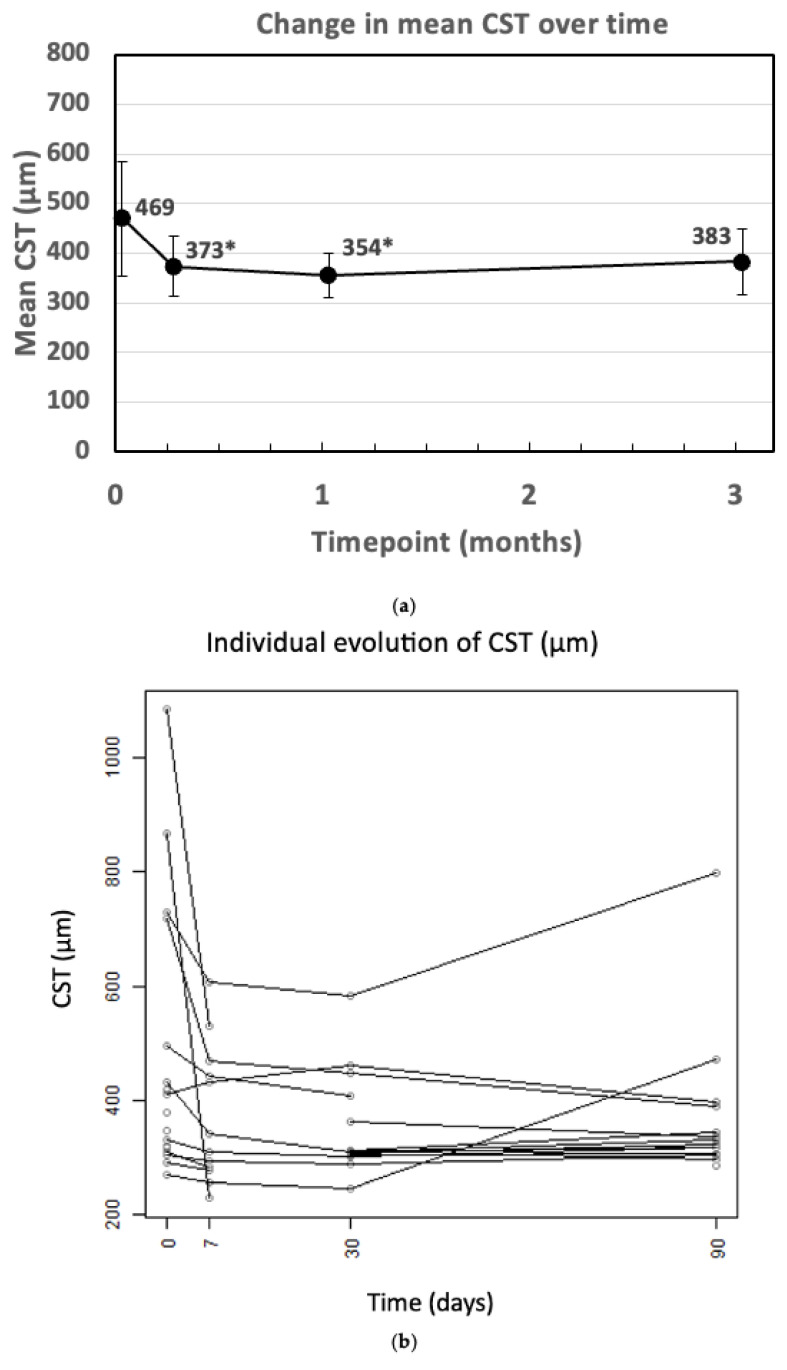
(**a**) Change in CST over time (* *p* < 0.05 vs. baseline). CST: central subfoveal thickness m: month; w: week. (**b**) The individual evolution of CST.

Compared to a mean baseline value of 469.4 ± 53.8 µm, CST significantly decreased by 20% to 373.1 ± 34.7 µm at 1 week (*p* = 0.002) and to 354.4 ± 24.1 µm at 1 month (*p* = 0.011). A decreased CST was also documented at 3 months (382.1 ± 34.2 µm); however, the difference from baseline did not reach statistically significance (*p* = 0.08). Figure 1a depicts the change in CST over the different time points. Figure 1b illustrates the individual evolution of CST.

**Figure 2:**
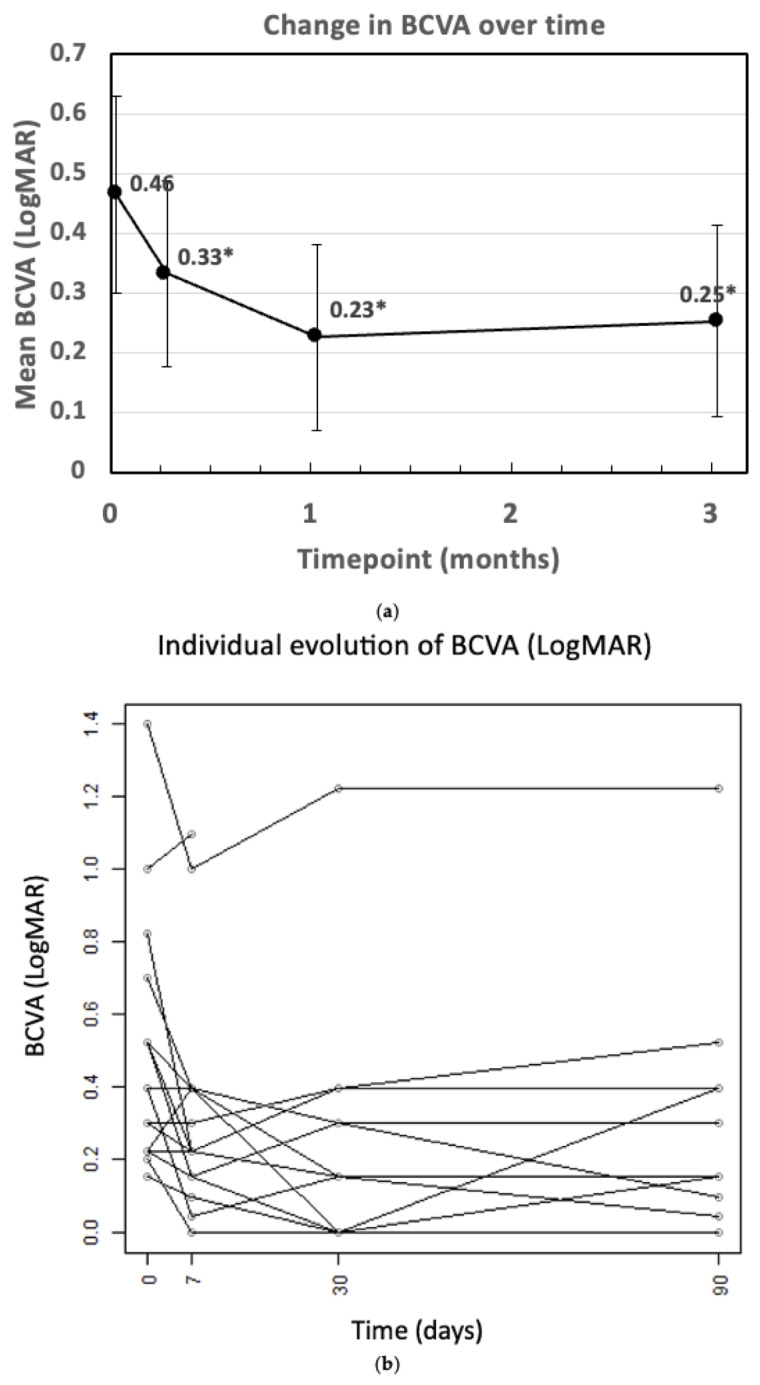
(**a**) Change in BCVA over time (* *p* < 0.05 vs. baseline). BCVA: best corrected visual acuity; m: month; w: week. (**b**) The individual evolution of BCVA.

Similarly, BCVA consistently improved compared to baseline (Figure 2a). Specifically, the decrease in BCVA LogMAR indicated an improvement in visual acuity over time. Compared to a baseline BCVA (in LogMAR) of 0.47, the mean BCVA decreased significantly to 0.33 at 1 week (*p* = 0.001), 0.23 at 1 month (*p* < 0.001), and 0.25 at 3 months (*p* < 0.001). Figure 2b illustrates the individual evolution of BCVA.

**Figure 3:**
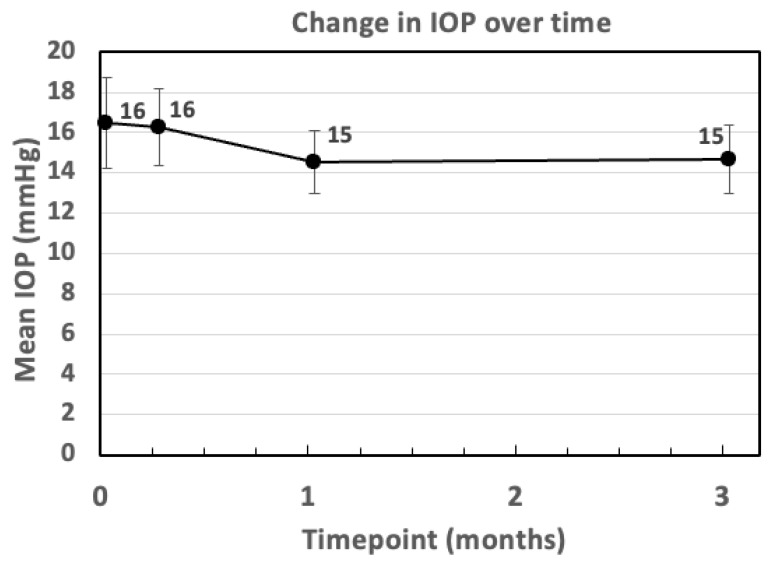
Change in IOP over time. IOP: intraocular pressure; m: month; w: week.

When examining changes in IOP, no significant differences were documented during follow-up assessments (Figure 3). Notably, one eye (5.6%) developed ocular hypertension, which was treated with antihypertensive drops. Mean values for CST, BCVA and IOP over time are reported in Table 2.

### Correlation Between OCT Biomarkers and Treatment Response

**Figure 4:**
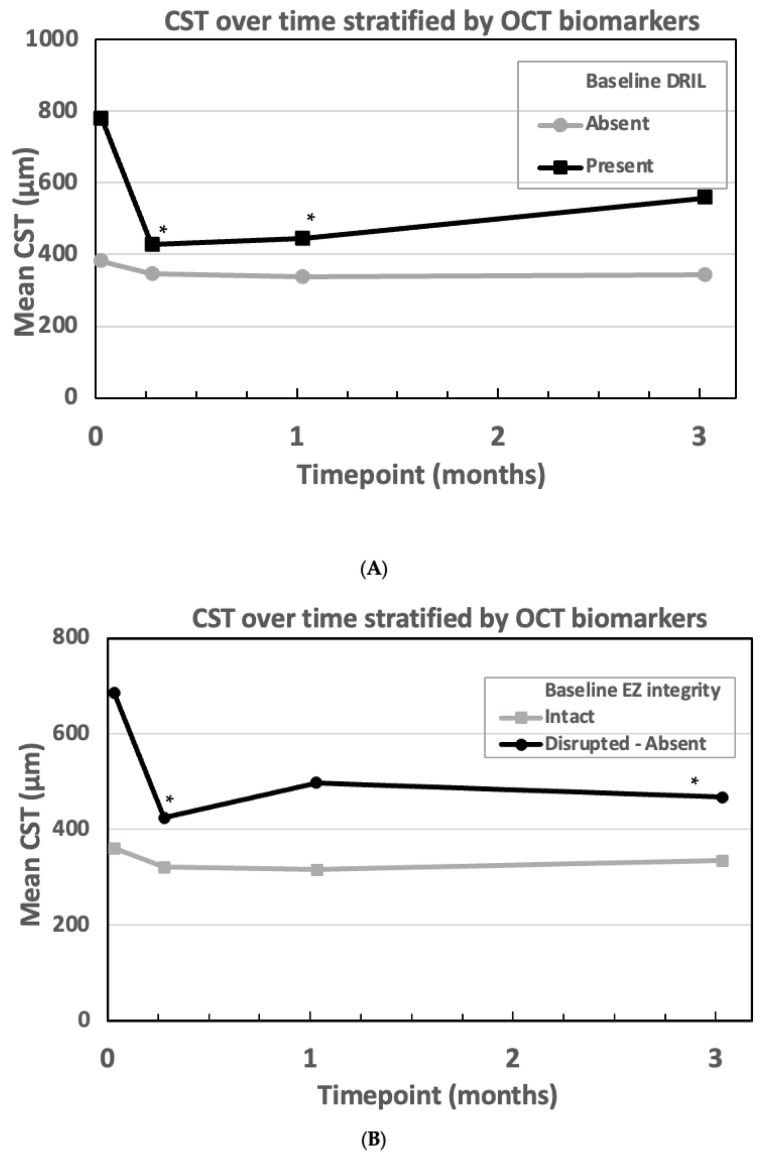
(**A**,**B**) CST over time stratified by OCT biomarkers (Top Panel: Baseline DRIL; Bottom Panel: Baseline EZ Integrity) (* *p* < 0.05 vs. baseline). CST: central subfoveal thickness; DRIL: disorganization of the retinal inner layer; EZ: ellipsoid zone; m: month; OCT: optical coherence tomography; w: week.

We assessed the correlation between two OCT biomarkers and the response to treatment (Figure 4A,B). Specifically, we categorized patients according to two different OCT features at baseline: presence or absence of DRIL and EZ integrity (intact versus disrupted/absent). The biomarkers at baseline are reported in Table 3. The presence of a disrupted/absent EZ at baseline was associated with a reduction in CST that resulted statistically significant at 1 week- and 3 month-timepoints (*p* = 0.004 and 0.038, respectively). No significant differences were documented during follow-up in those patients with intact EZ integrity (Figure 4A). The presence of DRIL at baseline was associated with a significant reduction in CST at 1 week (*p* < 0.001) and 1 month (*p* = 0.009). Figure 4B demonstrates the change in CST over time in patients categorized according to OCT biomarkers (DRIL and EZ/ELM integrity). Furthermore, the baseline CST was identified as a predictor at 1 week (*p* < 0.001), 1 month (*p* = 0.001), and 3 months (*p* = 0.003). Higher baseline CST is associated with a larger reduction in CST. Conversely, baseline HRF (*p* = 0.437) and baseline HbA1c (*p* = 0.512) did not show significant effects on CST changes over time.

## 4. Discussion

During our study we examined the therapeutic impact of DEX-I, administered alongside cataract surgery, in patients with pDME who were non-responders to anti-VEGF injections. These effects were evaluated in relation to predictive OCT biomarkers, including EZ integrity, DRIL, CST, HRF, as well as HbA1c at baseline.

Our findings showed significant anatomical (CST) and functional (BCVA) improvements promoted by the combined procedure. While the anatomical changes did not maintain statistical significance after 1 month, a statistically significant functional improvement (BCVA) persisted throughout the study follow-up. These results align with previous research indicating that DEX-I is an effective treatment for DME, particularly in cases where anti-VEGF treatments are unsuitable or ineffective [13,14,15,16]. DEX-I appears to be very effective, with some cases showing more evident benefits, mainly in eyes with moderate to severe DME [central retinal thickness (CRT) > 410 µm] and with chronic/persistent DME [3].

### 4.1. Comparing DEX-I with and Without Simultaneous Cataract Surgery

To assess whether additional cataract surgery has an effect in terms of anatomical and functional results, it is important to compare our findings with those of studies investigating DEX-I alone without simultaneous phacoemulsification. In a four-month prospective trial by Lazic et al., the efficacy of DEX-I was evaluated in patients with chronic DME unresponsive to three consecutive anti-VEGF injections [17]. The authors reported significant improvements in CST persisting after 3 months of follow-up, whereas our findings showed significant changes confirmed at 1 month but not at 3 months. It can be hypothesized that this discrepancy might be due to cataract surgery-induced cystoid macular edema, which usually develops 4 to 12 weeks postoperatively (with a peak expected around week 6) and could affect CST reduction at 3 months [18].

Regarding BCVA, Lazic et al. did not observe significant changes in BCVA at 1 and 3 months, whereas our study demonstrated significant improvements at all time points. This difference could be due to the simultaneous phacoemulsification: the removal of the cataract may lead to better functional outcomes.

Similar CST and BCVA trends were observed in another study by Bonfiglio et al., which analyzed the effects of DEX-I in patients with DME unresponsive to ranibizumab treatment, focusing on inflammatory OCT biomarkers [5].

Unlike previous studies, our observations need to be interpreted in light of the concomitant adoption of cataract surgery and DEX-I. Specifically, our findings seem to suggest that cataract removal contributes to sustained functional gains despite anatomical variability, thereby highlighting a divergence between anatomical and functional outcomes. This could be attributed to cataract surgery-induced inflammation, which paradoxically may enhance visual function without a corresponding reduction in CST. Although DEX-I is known to mitigate post-surgical inflammation, its anti-inflammatory effects may contribute to functional improvement but not counteract anatomical changes over three months. The observed discrepancy aligns with the known weak correlation between CST and BCVA, which often diminishes with prolonged treatment and underline the complex interplay of anatomical and functional factors in determining patient outcomes [19].

### 4.2. OCT Biomarkers and Their Role in Treatment Response

Consistent with previous studies, our analysis found that disrupted or absent EZ, DRIL, and higher baseline CST significantly predicted favorable anatomical outcomes [3,8]. These OCT features are indicative of chronicity and are associated with better responses to corticosteroids compared to anti-VEGF therapies in DME patients. A commonly described feature in this context is the presence of hyperreflective foci (HRF). The exact origin of this entity is unknown. In DME it is thought to be activated resident microglial cells, initially located close to ganglion cells and other layers of the inner retina. This is supported by the finding that CD14, released by activated microglial cells, is increased in the aqueous humor in the presence of HRF [8].

It is interesting to note that baseline HRF did not significantly influence CST over time in our study. Previous research has highlighted HRF as a marker of disease severity and a potential predictor of corticosteroid response, however the results in the literature are controversial [7,20]. Several studies have reported a significant correlation between baseline HRF and treatment outcomes—both anatomical and functional—whereas others, including our findings, suggest that baseline HRF is not a strong predictor of response after treatment. Nevertheless, a consistent observation across the literature is that the number of HRF tends to decrease significantly following treatment [20].

The absence of statistically significant findings in our study may be related to the way HRF was classified. Treating HRF as a continuous rather than a categorical variable may provide more precise results. Additionally, manual HRF quantification in OCT images is time-consuming and prone to variability. With rapid advances in computer science, there is great potential for automated quantification of HRF [21,22].

By integrating artificial intelligence into the analysis of OCT images, both clinical and research settings could benefit from improved efficiency and consistency. Despite the development of several algorithms aimed at detecting hyperreflective lesions in B-scan images, their performance remains inconsistent. This may be due to the small size and subtle appearance of these lesions, suggesting that further refinement is needed [23].

It is important to note that AI in diabetic retinopathy (DR) has already made progress, particularly in lesion segmentation. Several AI models have been developed for this purpose in recent years. These include convolutional neural networks (CNNs), which have performed well in extracting features and handling variability in image quality. A technique that applies deep CNN is the recent version of You Only Look Once (YOLO). It can extract features representing the severity of diabetic retinopathy within a short period of time [24]. Generative Adversarial Networks (GANs) have also been used. In addition, more targeted approaches, such as ResNet-based subnetworks were designed to highlight specific lesions. These methods have primarily focused on segmentation and quantification of typical DR-related changes such as microaneurysms, hard exudates, hemorrhages, and cotton-wool spots [23].

Nonetheless, incorporating AI into clinical workflows also raises important considerations, including data privacy, regulatory compliance, and compatibility with existing healthcare systems.

### 4.3. HbA1c and Treatment Response

Our analysis revealed no significant correlation between HbA1c levels and post-procedural CST changes. While poor glycemic control is associated with increased DME incidence and progression, its influence on treatment success is less clear [25,26]. Retrospective studies have shown that patients with HbA1c levels above 7% tend to show less improvement in BCVA and CST after treatment [27,28]. However, the prospective, randomized, phase 3 RIDE and RISE studies, which included 759 participants, found no significant differences in BCVA gain or CST reduction between patients with HbA1c levels above 7% and those below 7%. Notably, participants in the RIDE and RISE studies had reasonably well-controlled HbA1c levels, averaging around 7.7% over 24 months [29]. In our study, the mean baseline HbA1c was 7.5 ± 1.1%. The exclusion of patients with severely uncontrolled diabetes may have minimized the impact of glycemic control on treatment outcomes, representing a potential limitation. Future research should consider not only baseline HbA1c levels but also changes in metabolic control following treatment to clarify this relationship.

### 4.4. Limitations

Our study has several limitations, which need to be acknowledged. (1) The small sample size, retrospective design and completely male population limit the generalizability of our findings. (2) The inclusion of both eyes from some patients could have introduced correlated responses, potentially biasing results. (3) A lack of a control group further limits the ability to isolate the effects of combined cataract surgery and DEX-I treatment. (4) The follow-up duration was limited to 3 months. Future research should focus on longer follow-up periods to better understand the long-term effects of this combined approach. (5) Investigating additional predictive biomarkers, particularly with advanced imaging technologies, may refine personalized treatment strategies for DME.

## 5. Conclusions

Our findings suggest that DEX-I remains effective and safe in patients with pDME undergoing cataract surgery. Our findings seem to suggest that cataract removal contributes to sustained functional gains despite anatomical variability. Patients with OCT features indicative of chronicity and severity (e.g., DRIL, disrupted EZ and higher CST) at baseline may be better candidates for the combination of dexamethasone and cataract surgery. OCT biomarkers may provide valuable insights into treatment response, highlighting the importance of personalized approaches.

## Figures and Tables

**Table 1:** Baseline Characteristics. DM: diabetes mellitus; HbA1c: Hemoglobin A1c.

Variables	Mean ± SD or N (%)
Age (years)	62.7 ± 9.7
Male Gender	11 (100%)
History of diabetes (years)	19.4 ± 9.1
DM Type 1/Type 2	3 (17%)/15 (83%)
HbA1c (%)	7.5 ± 1.1
Systolic Blood Pressure (mmHg)	127.6 ± 13.1
Diastolic Blood Pressure (mmHg)	71.0 ± 8.9

**Table 2:** Mean values with standard deviation for CST, BCVA and IOP over time. CST: central subfoveal thickness; BCVA: best corrected visual acuity; IOP: intraocular pressure.

	Baseline	1 Week	1 Month	3 Months
**CST (µm)**	469.44 ± 228.31	373.08 ± 120.33	354.36 ± 90.31	383.07 ± 132.60
**BCVA (LogMAR)**	0.46 ± 0.33	0.33 ± 0.31	0.23 ± 0.31	0.25 ± 0.32
**IOP (mmHg)**	16.47 ± 4.53	16.26 ± 3.61	14.53 ± 2.85	14.67 ± 3.09

**Table 3:** Functional and morphological biomarkers at baseline. CST: central subfoveal thickness; BCVA: best corrected visual acuity; DRIL: disorganization of the retinal inner layer; EZ: ellipsoid zone; ELM: external limiting membrane; HRF: hyperreflective foci.

Baseline Variables	Mean (±SD) or N (%)
BCVA (LogMAR)	0.46 (±0.33)
CST (µm)	469.4 (±228.31)
EZ/ELM	
- Intact	12 (66.7%)
- Disrupted/Absent	6 (33.3%)
DRIL	
- Absent	14 (77.8%)
- Present	4 (22.2%)
HRF (1 missing)	
- Less than 30	6 (33.3%)
- More than 30	11 (61.1%)

## Data Availability

The original contributions presented in this study are included in the article. Further inquiries can be directed to the corresponding author.

## Abbreviations

The following abbreviations are used in this manuscript:

BCVA	best-corrected visual acuity
CRT	central retinal thickness
CST	central subfoveal thickness
DDM	diabetes mellitus
DEX-I	dexamethasone intravitreal implant
DRIL	disorganization of the retinal inner layers
ELM	external limiting membrane
ETDRS	early treatment diabetic retinopathy sstudy
EZ	ellipsoid zone
HRF	hyperreflective foci
IOP	intraocular pressure
VEGF	vascular endothelial growth factor
